# miR-146a promotes the initiation and progression of melanoma by activating Notch signaling

**DOI:** 10.7554/eLife.01460

**Published:** 2014-02-18

**Authors:** Matteo Forloni, Shaillay Kumar Dogra, Yuying Dong, Darryl Conte, Jianhong Ou, Lihua Julie Zhu, April Deng, Meera Mahalingam, Michael R Green, Narendra Wajapeyee

**Affiliations:** 1 Department of Pathology, Yale University School of Medicine, New Haven, United States; 2 Singapore Institute of Clinical Sciences, Agency for Science Technology and Research (A*STAR), Singapore, Singapore; 3 Program in Molecular Medicine, University of Massachusetts Medical School, Worcester, United States; 4 Program in Gene Function and Expression, University of Massachusetts Medical School, Worcester, United States; 5 Programs in Gene Function and Expression, Molecular Medicine, and Bioinformatics and Integrative Biology, University of Massachusetts Medical School, Worcester, United States; 6 Department of Pathology, University of Massachusetts Medical School, Worcester, United States; 7 Dermatopathology Section, Department of Dermatology, Boston University School of Medicine, Boston, United States; 8 Program in Gene Function and Expression, Howard Hughes Medical Institute, University of Massachusetts Medical School, Worcester, United States; Fred Hutchinson Cancer Research Center, United States

**Keywords:** miRNA, BRAF, NRAS, melanoma, human

## Abstract

Oncogenic mutations in BRAF and NRAS occur in 70% of melanomas. In this study, we identify a microRNA, miR-146a, that is highly upregulated by oncogenic BRAF and NRAS. Expression of miR-146a increases the ability of human melanoma cells to proliferate in culture and form tumors in mice, whereas knockdown of miR-146a has the opposite effects. We show these oncogenic activities are due to miR-146a targeting the NUMB mRNA, a repressor of Notch signaling. Previous studies have shown that pre-miR-146a contains a single nucleotide polymorphism (C>G rs2910164). We find that the ability of pre-miR-146a/G to activate Notch signaling and promote oncogenesis is substantially higher than that of pre-miR-146a/C. Analysis of melanoma cell lines and matched patient samples indicates that during melanoma progression pre-miR-146a/G is enriched relative to pre-miR-146a/C, resulting from a C-to-G somatic mutation in pre-miR-146a/C. Collectively, our results reveal a central role for miR-146a in the initiation and progression of melanoma.

**DOI:** http://dx.doi.org/10.7554/eLife.01460.001

## eLife digest

Cancer is a leading cause of death worldwide, and although cancer can have many causes, mutations to a small number of genes are often responsible for a large number of cancer cases. For example, about 70% of cases of the deadliest form of skin cancer have mutations in two specific genes. Many cancer-causing genes are regulated by small RNA molecules that impede the function of other genes by blocking the machinery that turns a gene into a functional protein. However, until recently there was a limited understanding of the role of these microRNAs in the skin cancers caused by the most common mutations.

Now, Forloni et al. have looked at all the microRNAs present in cells carrying a mutated form of the one of these genes, and compared these with the microRNAs found in healthy cells. A microRNA, called miR-146a, was discovered to be much more common in the cancer cells. Moreover, production of this microRNA was increased by the two cancer-causing mutations. Forloni et al. found that increasing production of this microRNA caused human skin cancer cells to grow faster, and also caused tumors to develop in mice. Reducing the level of miR-146a had the opposite effect.

Forloni et al. also looked at cancer cells taken from individuals at different stages of skin cancer and found that, as the disease progresses, an unprocessed form of miR-146a tends to acquire a mutation, which leads to much higher levels of the active, processed form of miR-146a in the cancer cells. High levels of miR-146a also switched off a gene that encodes for a protein that, in turn, switches off a protein called NOTCH that is linked to skin cancer. As such, excess miR-146a actually increases the activation of the NOTCH protein.

These results led Forloni et al. to test if delivering drugs that block the production of miR-146a by inhibiting skin cancer-causing genes, along with other drugs that inhibit the Notch signaling pathway, could be more effective than treatment with either drug on its own. The combined treatment was very effective against human skin cancer cells and could represent a promising development in the treatment of this disease.

**DOI:** http://dx.doi.org/10.7554/eLife.01460.002

## Introduction

Melanoma is the deadliest form of skin cancer accounting for ∼80% of skin cancer-related deaths (Miller and Mihm, 2006). The most commonly observed oncogenic events in melanomas are activating mutations in the BRAF and NRAS proto-oncogenes, which occur in 70% of cases (Miller and Mihm, 2006; Tsao et al., 2012). Activating mutations in BRAF and NRAS genes cause constitutive activation of downstream signaling pathways, resulting in pro-proliferative and anti-apoptotic effects that promote cellular transformation, tumor growth and metastasis (Downward, 2003; Wellbrock et al., 2004; Karnoub and Weinberg, 2008).

Oncogenic BRAF mutants (typically BRAFV600E) primarily activate the MAPK pathway (Wellbrock et al., 2004). Genetic and pharmacological studies have shown that disruption of the BRAF-MEK-ERK pathway blocks the growth of melanoma cells harboring an oncogenic BRAF mutation and thus represents an attractive therapeutic target (Wellbrock et al., 2004; Miller and Mihm, 2006; Tsao et al., 2012). Oncogenic NRAS is capable of activating multiple downstream pathways, including BRAF-MEK-ERK, all of which are thought to play an important role in NRAS driven oncogenesis (Downward, 2003; Karnoub and Weinberg, 2008).

The Notch pathway is an evolutionary conserved signaling cascade that has an essential role in embryonic development and cell renewal in the adult (Guruharsha et al., 2012). Notch signaling has a key role in melanoblast and melanocyte homeostasis (Haass and Herlyn, 2005; Aubin-Houzelstein et al., 2008; Kumano et al., 2008). For example, conditional ablation of Notch signaling in the melanocyte lineage leads to drastic elimination of melanoblasts and melanocyte stem cells (Moriyama et al., 2006). NOTCH1 expression is normally decreased in mature melanocytes, whereas melanomas regain expression and activity of NOTCH1 (Balint et al., 2005; Pinnix et al., 2009). NOTCH1 is required for melanoma formation, can transform primary human melanocytes and can confer metastatic properties to primary melanoma cells (Liu et al., 2006; Asnaghi et al., 2012).

miRNAs are small non-coding RNAs that function by regulating the stability or translation of mRNAs (Bartel, 2004; He and Hannon, 2004; Leung and Sharp, 2006). miRNAs have been implicated in essentially all aspects of tumor biology including tumorigenesis, angiogenesis and metastasis (Croce, 2009; Garzon et al., 2009) indicating that, similar to protein-coding genes, miRNAs function as crucial regulators of tumor initiation and progression. Interestingly, miRNAs can act as either tumor suppressors or oncogenes depending on the functions of their targets (Croce, 2009; Garzon et al., 2009). High-throughput profiling has revealed dysregulation of miRNAs in a variety of cancers (Croce, 2009; Garzon et al., 2009). For example, more than half of miRNA genes in human cancers are located in chromosomal regions that frequently exhibit amplification, deletion, or translocation (Croce, 2009; Garzon et al., 2009).

Whether microRNAs (miRNAs) have a role in BRAF- and NRAS-driven melanoma initiation and progression remains to be determined. In this study, we perform small RNA profiling and identify miR-146a as an oncogenic BRAF- and NRAS-regulated miRNA that promotes the initiation and progression of melanoma. We show that miR-146a functions as an oncogene by activating Notch signaling, and that during melanoma progression pre-miR-146a can acquire a somatic mutation that enhances its oncogenic activity.

## Results

### Small RNA profiling identifies miR-146a as an oncogenic BRAF- and NRAS-regulated microRNA

**Figure 1.**
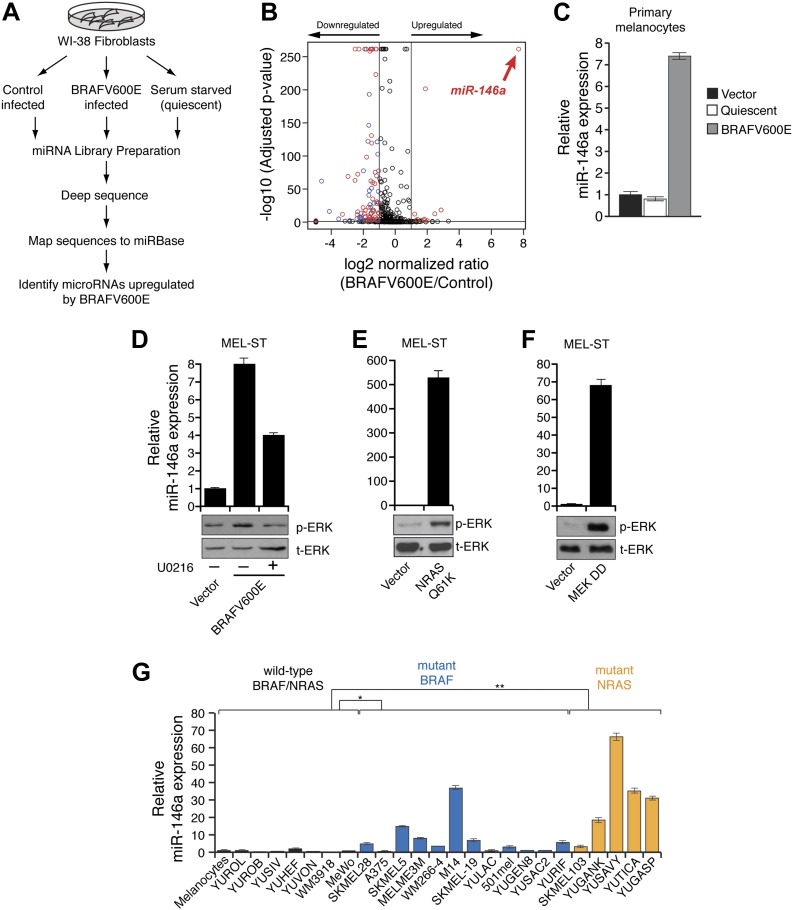
BRAFV600E and NRASQ61K upregulate miR-146a expression. (**A**) Schematic summary of study design. (**B**) Volcano plot showing miR-146a (red arrow) as the most upregulated miRNA. (**C**) qRT-PCR analysis of miR-146a expression in primary melanocytes transduced with BRAFV600E (gray) or Vector control (black) retrovirus particles. Quiescent cells were used as a control (light gray). (**D**) qRT-PCR analysis (top) measuring miR-146a expression in MEL-ST cells expressing BRAFV600E in the presence (+) or absence (−) of the MEK inhibitor U0216 (10 µM) relative to Vector control. Immunoblots (bottom) of phosphorylated (p-) ERK (upper) and total (t-) ERK (lower) show that BRAFV600E activates MEK-dependent phosphorylation of ERK. (**E**) qRT-PCR analysis (top) measuring miR-146a expression in MEL-ST cells expressing NRASQ61K relative to Vector control. Immunoblots (bottom) of phosphorylated (p-) ERK (upper) and total (t-) ERK (lower) show that NRASQ61K activates phosphorylation of ERK. (**F**) qRT-PCR analysis (top) measuring miR-146a expression in MEL-ST cells expressing constitutively active MEK (DD), relative to the Vector control. Immunoblots (bottom) of p-ERK (upper) and t-ERK (lower) show that MEK (DD) activates phosphorylation of ERK. (**G**) qRT-PCR analysis monitoring miR-146a expression in melanocyte and melanoma cell lines. BRAF (blue) and NRAS (orange) mutant cell lines are indicated. **DOI:** http://dx.doi.org/10.7554/eLife.01460.003

To identify possible BRAFV600E-regulated miRNAs, we generated miRNA libraries from primary lung fibroblast WI-38 cells transduced with retrovirus expressing BRAFV600E or an empty vector and performed deep sequencing analysis. To rule out miRNAs that are altered due to cell cycle arrest, we also sequenced a miRNA library generated using quiescent WI-38 cells (Figure 1A). Our analysis identified miR-146a as the most upregulated miRNA by BRAFV600E (Figure 1B). To confirm that miR-146a is a target of BRAFV600E, we transduced human fibroblasts WI-38, IMR-90 and primary human melanocytes (hereafter referred to as melanocytes) with retroviruses expressing BRAFV600E. We found that BRAFV600E activates miR-146a expression in WI-38 as well as in IMR-90 and melanocytes (Figure 1—figure supplement 1, Figure 1C).

**Figure 1—figure supplement 1.**
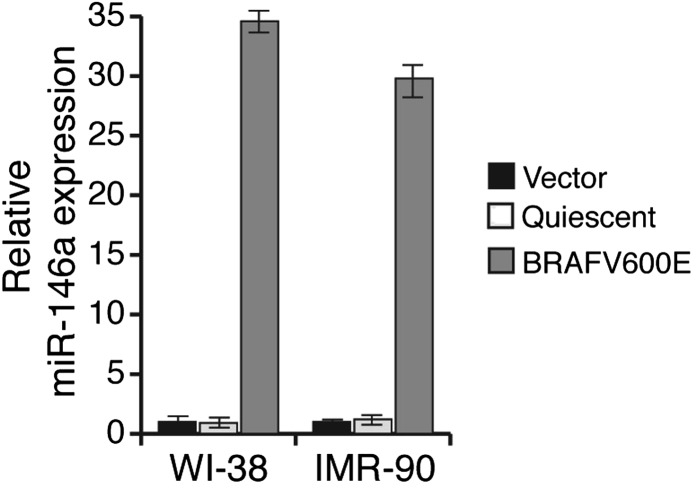
Ectopic expression of BRAFV600E in WI-38 and IMR-90 cells stimulates miR-146a expression. qRT-PCR analysis of miR-146a expression in WI-38 or IMR-90 cells transduced with BRAFV600E (gray) or Vector control (black) retrovirus particles. Quiescent cells were used as a control (light gray). **DOI:** http://dx.doi.org/10.7554/eLife.01460.004

**Figure 1—figure supplement 2.**
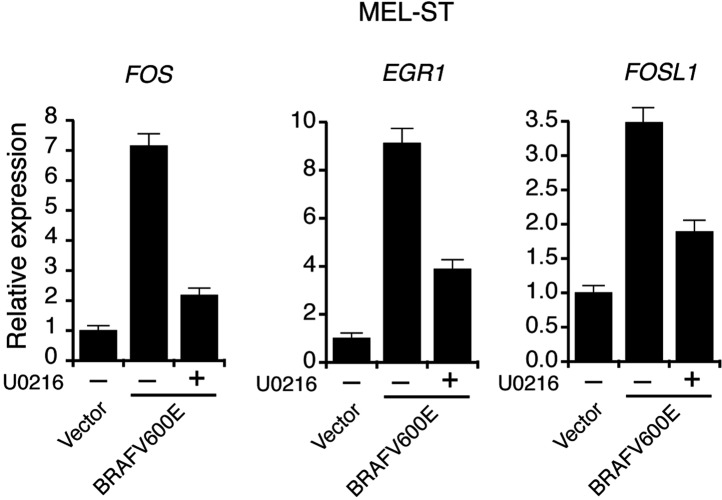
Activation of MAP Kinase pathway target genes upon ectopic expression of BRAFV600E in MEL-ST cells. qRT-PCR analysis of MEK-ERK transcriptional targets *FOS*, *EGR1* and *FOSL1* under indicated conditions in MEL-ST cells expressing either an empty vector or BRAFV600E. **DOI:** http://dx.doi.org/10.7554/eLife.01460.005

**Figure 1—figure supplement 3.**
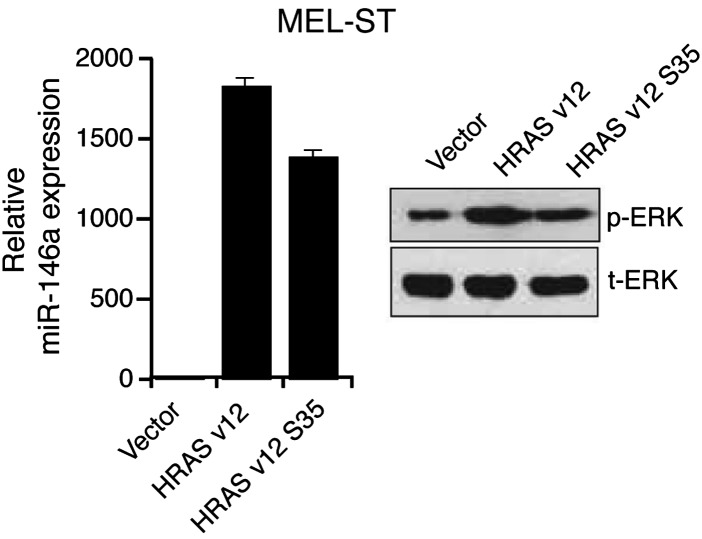
Ectopic expression of HRAS v12 or HRAS v12 S35 in MEL-ST cells stimulates miR-146 expression. qRT-PCR analysis of miR-146a (left) and immunoblot analysis (right) of phosphorylated (p-) ERK, total (t-) ERK in MEL-ST cells transduced with empty vector, HRAS v12, or HRAS v12s35. **DOI:** http://dx.doi.org/10.7554/eLife.01460.006

**Figure 1—figure supplement 4.**
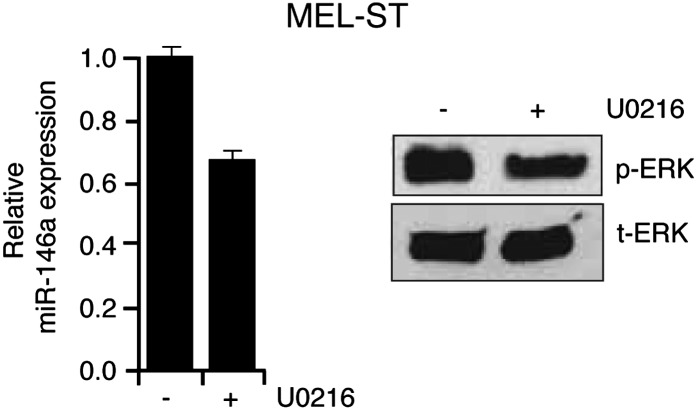
Inhibition of MAP kinase signaling by MEK inhibitor blocks HRAS v12-mediated miR-146a upregulation. qRT-PCR analysis (left) to monitor miR-146a expression and immunoblot analysis (right) of phosphorylated (p-) ERK and total (t-) ERK in MEL-ST cells transduced with HRAS v12 in the presence (+) or absence (−) of the MEK inhibitor U0216. **DOI:** http://dx.doi.org/10.7554/eLife.01460.007

**Figure 1—figure supplement 5.**
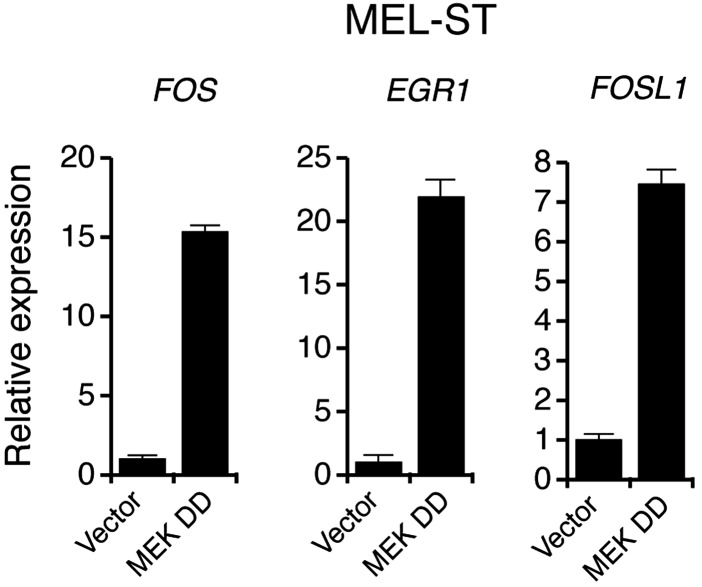
Ectopic expression of MEK DD stimulates the transcription of MAP kinase target genes. qRT-PCR analysis of MEK-ERK transcriptional targets *FOS*, *EGR1* and *FOSL1* in MEL-ST cells expressing either a empty vector or constitutively active MEK (MEK DD). **DOI:** http://dx.doi.org/10.7554/eLife.01460.008

Next, we asked whether BRAF-MEK-ERK signaling is required for miR-146a upregulation. We stably expressed BRAFV600E or an empty vector in MEL-ST cells, which are immortalized melanocytes that have basal levels of BRAF-MEK-ERK signaling and can be transformed by single oncogenes (Gupta et al., 2005). Figure 1D shows that, as in melanocytes, introduction of BRAFV600E into MEL-ST cells upregulated miR-146a expression. To confirm that miR-146a upregulation requires BRAF-MEK-ERK signaling, we treated MEL-ST/BRAFV600E cells with the MEK inhibitor U0216. Figure 1D shows that treatment of MEL-ST/BRAFV600E cells with U0216 resulted in decreased miR-146a expression. As expected, transcriptional targets of the MAP kinase pathway were also downregulated following U0216 treatment (Figure 1—figure supplement 2). Similar to BRAFV600E, stable expression of NRASQ61K, HRAS v12 or a mutant that selectively activates BRAF-MEK-ERK signaling also resulted in increased miR-146a levels (Figure 1E, Figure 1—figure supplement 3), which was attenuated by addition of U0216 (Figure 1—figure supplement 4).

To further confirm that increased BRAF-MEK-ERK signaling is sufficient to induce miR-146a expression, we stably expressed a constitutively active MEK derivative (MEK DD) in MEL-ST cells. Figure 1F shows that expression of MEK DD substantially increased BRAF-MEK-ERK signaling and stimulated miR-146a expression. As expected, transcriptional targets of the MAP kinase pathway were also upregulated in cells expressing MEK DD (Figure 1—figure supplement 5).

Over 60% of melanomas harbor mutations in the BRAF and NRAS genes (Davies et al., 2002; Chin et al., 2006). We therefore asked whether miR-146a expression is upregulated in melanoma cell lines containing mutant BRAF or NRAS. We monitored miR-146a expression in a panel of BRAF/NRAS wild-type or BRAF or NRAS mutant melanoma cell lines, short-term patient-derived melanoma cultures and melanocytes. Figure 1G shows that miR-146a expression was significantly higher in a subset of BRAF and NRAS mutant melanoma cell lines compared to both BRAF/NRAS wild-type melanoma cell lines and melanocytes. Collectively, our results show that BRAF-MEK-ERK signaling is necessary and increased BRAF-MEK-ERK signaling is sufficient for upregulation of miR-146a.

**Figure 2.**
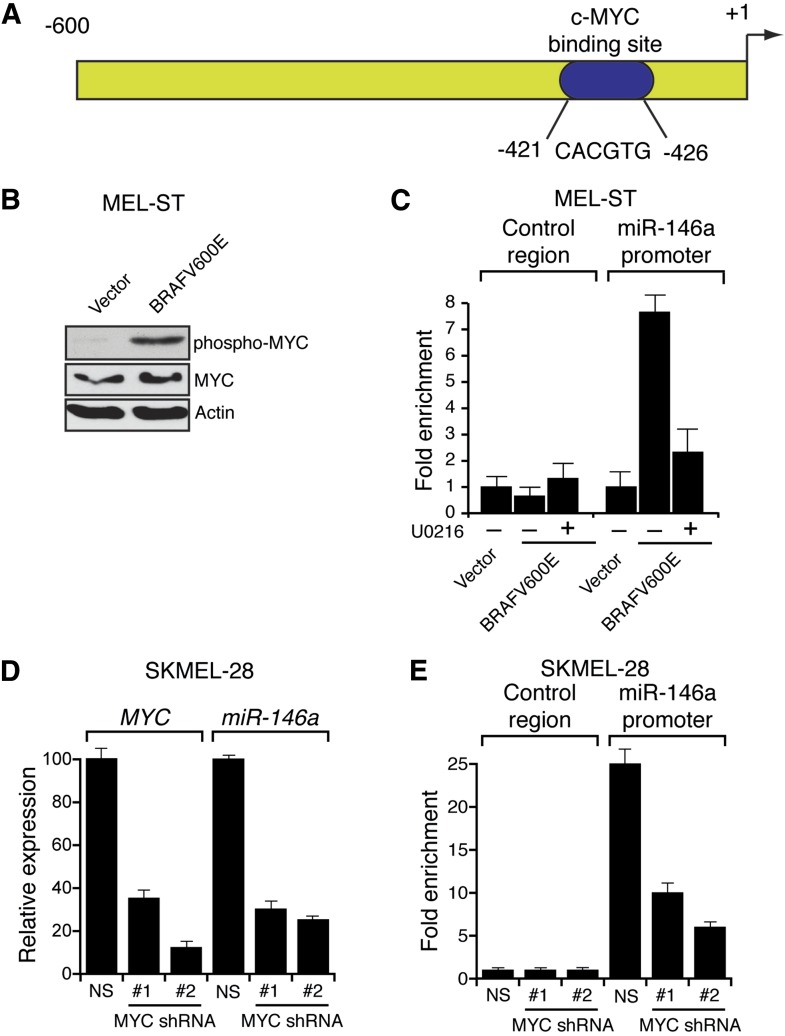
BRAFV600E upregulates miR-146a through MYC oncogene. (**A**) Schematic representation of MYC promoter and the miR-146a binding site that has been identified by PROMO analysis software. ‘+1’ indicates the transcription start site. (**B**) Immunoblot analysis of p-MYC and total MYC in whole cell lysates of MEL-ST cells transduced with empty vector or BRAFV600E. Actin was used as a loading control. (**C**) Chromatin immunoprecipitation (ChIP) assay measuring MYC binding to the miR-146a promoter in MEL-ST cells stably expressing BRAFV600E in the presence (+) or absence (−) of the MEK inhibitor U0216 relative to cells transduced with the empty vector. A non-specific control region served as a negative control for MYC recruitment. (**D**) qRT-PCR analysis of *MYC* mRNA (left) and miR-146a (right) expression in SKMEL-28 cells infected with *MYC* shRNAs. (**E**) qPCR analysis of MYC ChIP of miR-146a promoter and negative control in SKMEL-28 cells infected with *MYC* shRNAs. **DOI:** http://dx.doi.org/10.7554/eLife.01460.009

Several previous studies have shown that the BRAF-MEK-ERK pathway regulates transcription, protein stability and activity of MYC (Sears et al., 1999, 2000; Pintus et al., 2002; Marampon et al., 2006; Tsai et al., 2012). Analysis of the miR-146a promoter sequence using the PROMO 3.0 bioinformatics program (Messeguer et al., 2002) identified a potential MYC binding site (Figure 2A), suggesting that MYC promotes miR-146a upregulation. Stable expression of BRAFV600E in MEL-ST cells led to increased MYC expression, increased MYC phosphorylation (Figure 2B) and, as monitored in a chromatin immunoprecipitation (ChIP) assay, binding of MYC to the miR-146a promoter (Figure 2C). Notably, binding of MYC to miR-146a promoter was inhibited by the treatment of cells with U0216 (Figure 2C). Furthermore, shRNA-mediated knockdown of *MYC* in melanoma cell lines, SKMEL-28 and M14, substantially decreased miR-146a levels and the expression of other MYC target genes (Figure 2D, Figure 2—figure supplements 1–3). As expected, shRNA-mediated knockdown of *MYC* also resulted in decreased binding of MYC to the miR-146a promoter (Figure 2E). In addition to MYC, we also identified binding sites for transcription factors ETS1, ELK1, NF-κB, and c/EBPβ in the miR-146a promoter. However, unlike MYC, shRNA-mediated knockdown of these other transcription factors did not significantly affect miR-146a expression (Figure 2—figure supplement 4). Collectively, our results show that increased BRAF-MEK-ERK signaling results in activation and recruitment of MYC to the miR-146a promoter, which stimulates miR-146a transcription.

**Figure 2—figure supplement 1.**
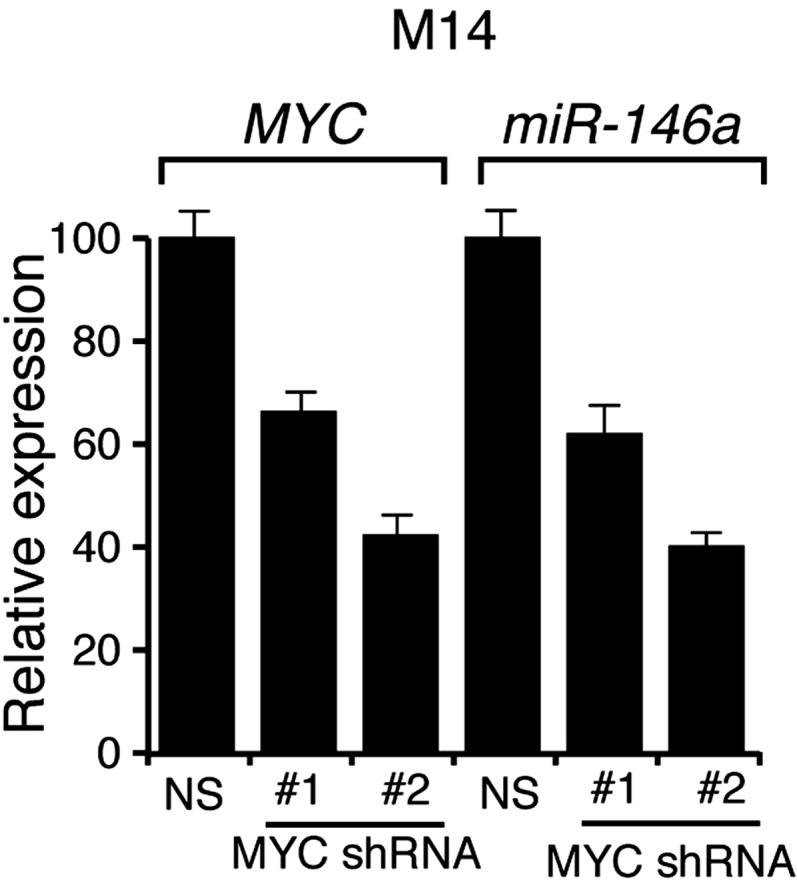
Analysis of MYC and miR-146a expression in M14 cells expressing shRNAs against MYC. qRT-PCR analysis of *MYC* and miR-146a expression in M14 cells transduced with *MYC* shRNA expression vectors relative to cells transduced with a non-specific (NS) shRNA vector. **DOI:** http://dx.doi.org/10.7554/eLife.01460.010

**Figure 2—figure supplement 2.**
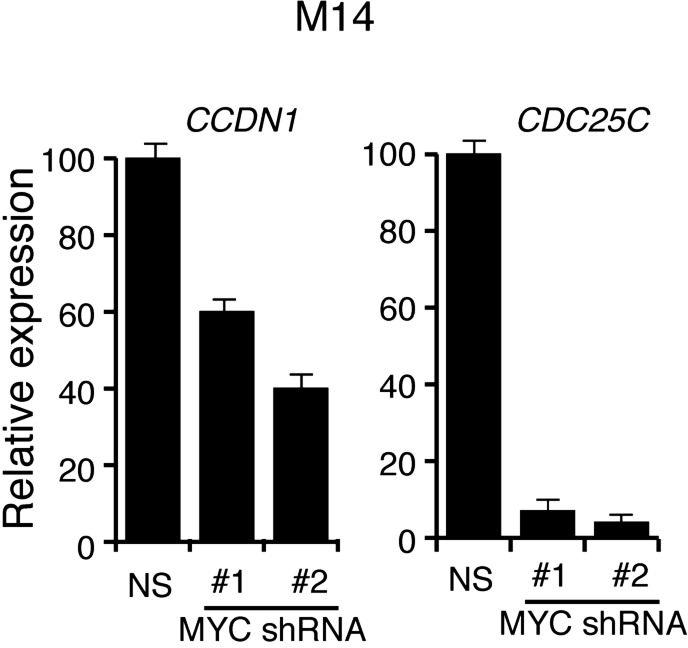
shRNA-mediated downrgulation of MYC in M14 cells inhibits the expression of MYC transcriptional target genes. qRT-PCR analysis of MYC targets *CCDN1* and *CDC25C* in M14 cells transduced with *MYC* shRNA expression vectors relative to cells transduced with a non-specific (NS) shRNA vector. **DOI:** http://dx.doi.org/10.7554/eLife.01460.011

**Figure 2—figure supplement 3.**
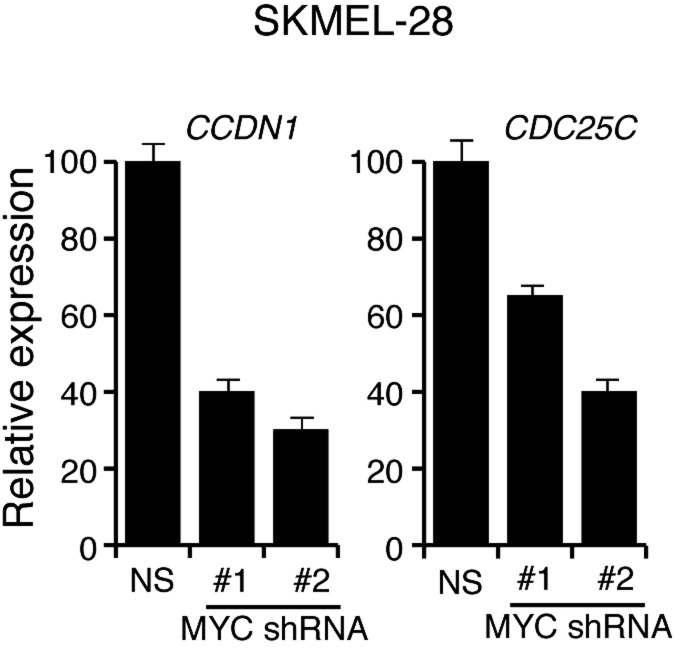
shRNA-mediated downrgulation of MYC in SKMEL-28 cells inhibits the expression of MYC transcriptional target genes. qRT-PCR analysis of MYC targets *CCDN1* and *CDC25C* in SKMEL-28 cells transduced with *MYC* shRNA expression vectors relative to cells transduced with a non-specific (NS) shRNA vector. **DOI:** http://dx.doi.org/10.7554/eLife.01460.012

**Figure 2—figure supplement 4.**
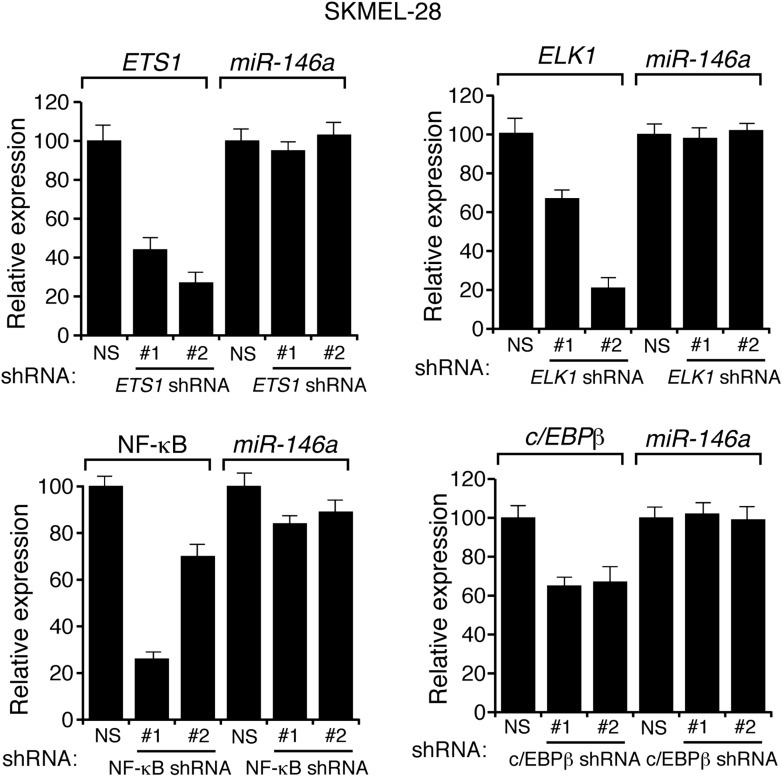
Transcriptional regulation of miR-146a. qRT-PCR analysis of ETS1, ELK1, NF-κB, c/EBPβ and miR-146a in SKMEL-28 expressing indicated shRNA. **DOI:** http://dx.doi.org/10.7554/eLife.01460.013

### miR-146a promotes the initiation and progression of melanoma

**Figure 3.**
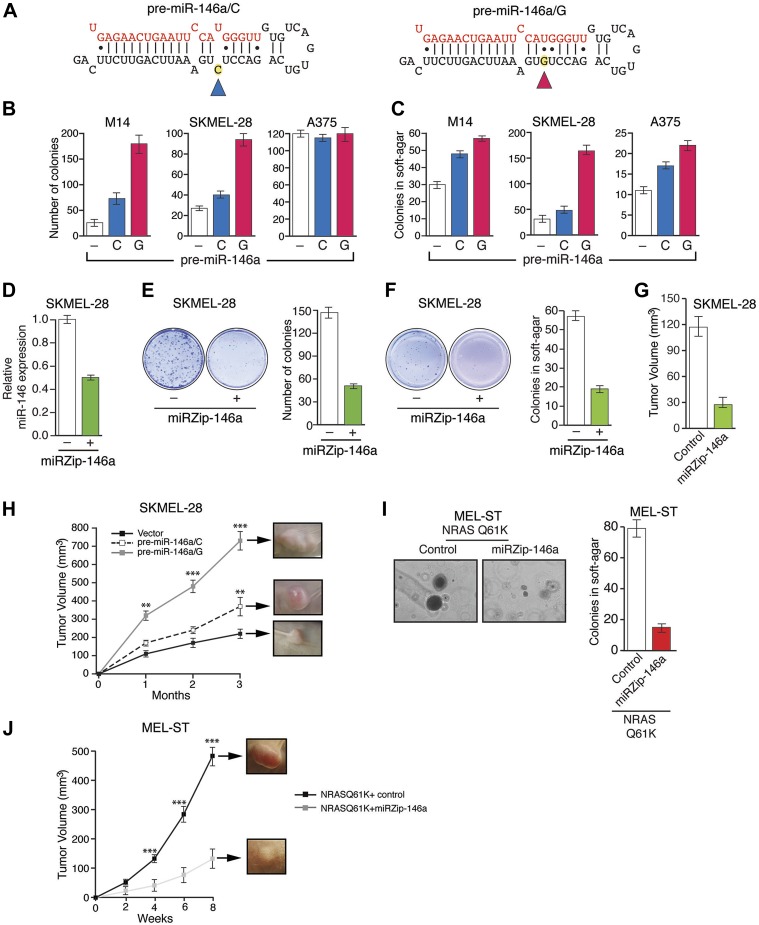
Oncogenic activity of pre-miR-146a/C and pre-miR-146a/G. (**A**) Schematic representation of pre-miR-146a/C and pre-miR-146a/G sequences. (**B** and **C**) Number of colonies formed in liquid (**B**) or soft-agar (**C**) by M14, SKMEL-28 or A375 melanoma cells expressing pre-miR-146a harboring a C or G at position 40, as compared to the Vector (−) control. Colonies were counted after 2 weeks (**B**) or 4 weeks (**C**) of growth. (**D**) qRT-PCR analysis of miR-146a expression in SKMEL-28 cells infected with miRZip-146a (+) or an empty vector (−). (**E** and **F**) Number of colonies formed in liquid (**E**) or soft-agar (**F**) by SKMEL-28 expressing miRZip-146a (+) or the vector (−) control. Colonies were counted after 2 weeks (**E**) or 4 weeks (**F**) of growth. (**G**) Average tumor volumes at 1 month time points for mice injected with SKMEL-28 expressing a control miRZip or miRZip-146a. (**H**) Average volume of tumors formed by SKMEL-28 cells (2.5 × 10^6^) expressing pre-miR-146a/C or pre-miR-146a/G, as compared to the Vector control, injected subcutaneously into the flanks of nude mice (n = 5). (**I**) Representative images (left) and colony number in soft-agar (right) for the NRASQ61K transformed MEL-ST cells that express an empty vector or miRZip-146a. (**J**) Average tumor volumes at the indicated time points for mice injected with NRASQ61K transformed MEL-ST cells expressing a control miRZip or miRZip-146a. **DOI:** http://dx.doi.org/10.7554/eLife.01460.014

Several recent studies have found that a pre-miR-146a SNP (C>G rs2910164) alters the expression of mature miR-146a and correlates with an increased risk to several cancers (Jazdzewski et al., 2008; Hezova et al., 2012; Hung et al., 2012; Lung et al., 2012; Wang et al., 2012; Yamashita et al., 2013). This SNP has been shown to occur in the pre-miR-146a sequence and does not alter the sequence of mature miR-146a (Jazdzewski et al., 2008; Hezova et al., 2012; Hung et al., 2012; Lung et al., 2012; Wang et al., 2012; Yamashita et al., 2013). The mechanism by which this SNP promotes tumorigenesis and its potential role in melanomagenesis remain to be determined. To address this question, we expressed both pre-miR-146a/C and pre-miR-146a/G (Figure 3A) in highly-tumorigenic human melanoma cell lines that efficiently formed colonies in soft-agar and tumors in immunocompromised mice. Significantly, consistent with a previous report (Jazdzewski et al., 2008), the amount of mature miR-146a produced from pre-miR-146a/G was higher than that from pre-miR-146a/C (Figure 3—figure supplement 1). Ectopic expression of pre-miR-146a/G promoted proliferation at a higher rate than pre-miR-146a/C, as evidenced by increased colony formation and increased proliferation in two of the three melanoma cell lines analyzed (Figure 3B, Figure 3—figure supplement 2). We also compared the ability of pre-miR-146a/C and pre-miR-146a/G to promote anchorage-independent growth in soft-agar. Again, pre-miR-146a/G-stimulated colony formation more efficiently than pre-miR-146a/C (Figure 3C). Notably, although expression of pre-miR-146a/G in A375 cells did not increase proliferation in liquid culture (Figure 3B), it did increase colony formation in soft-agar (Figure 3C). Conversely, inhibition of miR-146a by miRZip-146a in SKMEL-28 and M14 cells reduced colony formation in liquid culture and soft-agar, and inhibited tumor formation in mice (Figure 3D–G and Figure 3—figure supplement 3). Similarly, expression of a miR-146a locked nucleic acid (LNA)-based antagomiR in SKMEL-28 and M14 cells reduced colony formation in liquid culture and soft-agar (Figure 3—figure supplements 4 and 5). By contrast, expression of a miR-146a antagomiR in YUSIV cells, which express low levels of miR-146a, did not significantly affect colony formation in either liquid culture or soft-agar (Figure 3—figure supplement 6).

**Figure 3—figure supplement 1.**
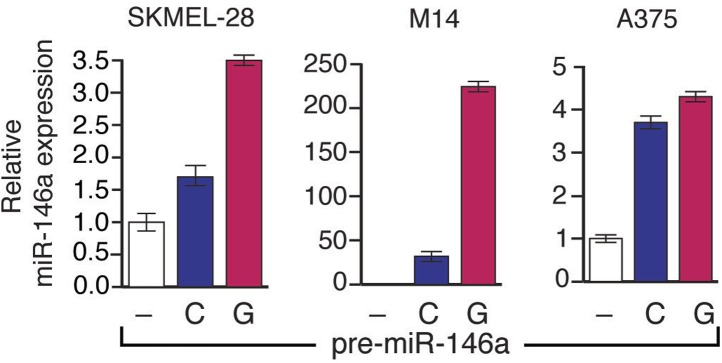
Analysis of miR-146a expression in SKMEL-28, A375 and M14 cells expressing either pre-miR-146a/G or pre-miR-146a/C construct. SKMEL-28, M14 and A375 cells stably expressing pre-miR-146a/C (C) or pre-miR-146a/G (G) or an empty vector (−) were analyzed for miR-146a expression by qRT-PCR. **DOI:** http://dx.doi.org/10.7554/eLife.01460.015

**Figure 3—figure supplement 2.**
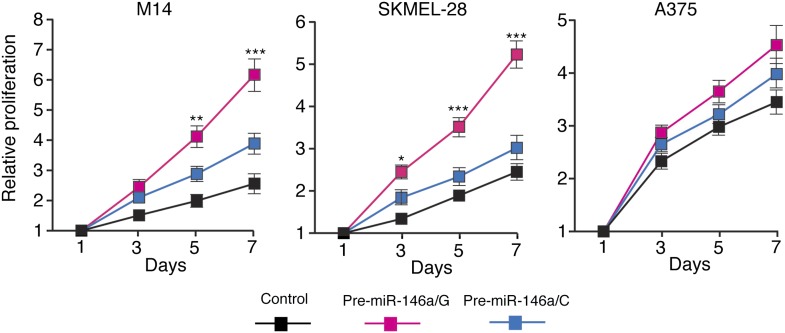
pre-miR-146a/G promotes proliferation of melanoma cells more effective than pre-miR-146a/C. M14, SKMEL-28 and A375 cells stably expressing pre-miR-146a/C (blue) or pre-miR-146a/G (red) or an empty vector (black) were analyzed for proliferation at indicated days. Relative proliferation is plotted. *, ** and *** represents p values <0.01, <0.001 and <0.0001 respectively. **DOI:** http://dx.doi.org/10.7554/eLife.01460.016

**Figure 3—figure supplement 3.**
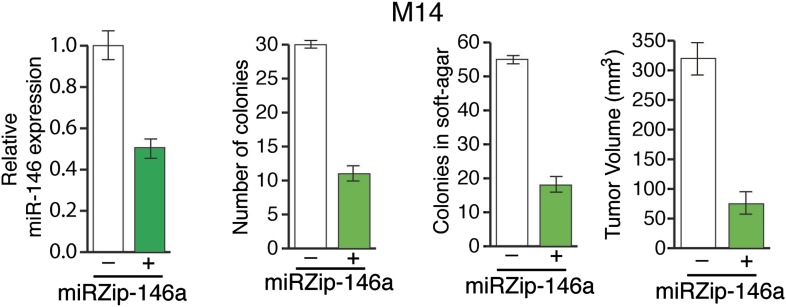
Inhibition of miR-146a expression blocks the proliferation and anchorage-independent growth of M14 cells. M14 cells either expressing a control miRZip vector or miRZip-146a were analyzed for miR-146a expression, colony formation, or growth in soft-agar or tumor formation in mice. Tumor volume at 1 month timepoint is plotted. **DOI:** http://dx.doi.org/10.7554/eLife.01460.017

**Figure 3—figure supplement 4.**
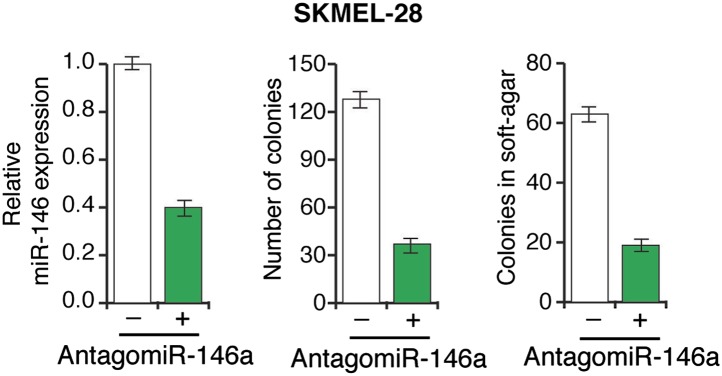
Inhibition of miR-146a expression blocks the proliferation and anchorage-independent growth of SKMEL-28 cells. SKMEL-28 cells either expressing a scrambled LNA-antagomiR or LNA-based miR146a antagomiR were analyzed for miR-146a expression (left), colony formation (middle) or growth in soft-agar (right). **DOI:** http://dx.doi.org/10.7554/eLife.01460.018

**Figure 3—figure supplement 5.**
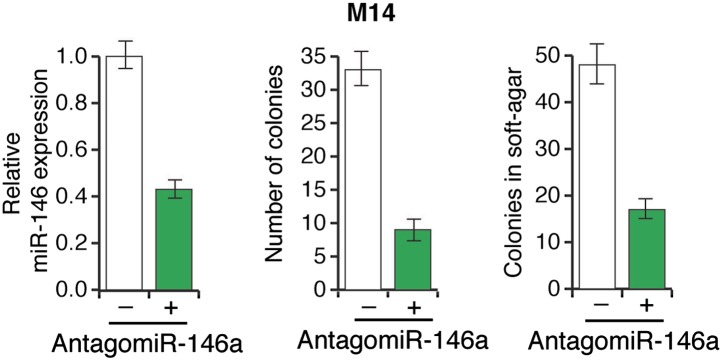
Inhibition of miR-146a expression blocks the proliferation and anchorage-independent growth of M14 cells. M14 cells either expressing a scrambled LNA-antagomiR or LNA-based miR146a antagomiR were analyzed for miR-146a expression (left), colony formation (middle) or growth in soft-agar (right). **DOI:** http://dx.doi.org/10.7554/eLife.01460.019

**Figure 3—figure supplement 6.**
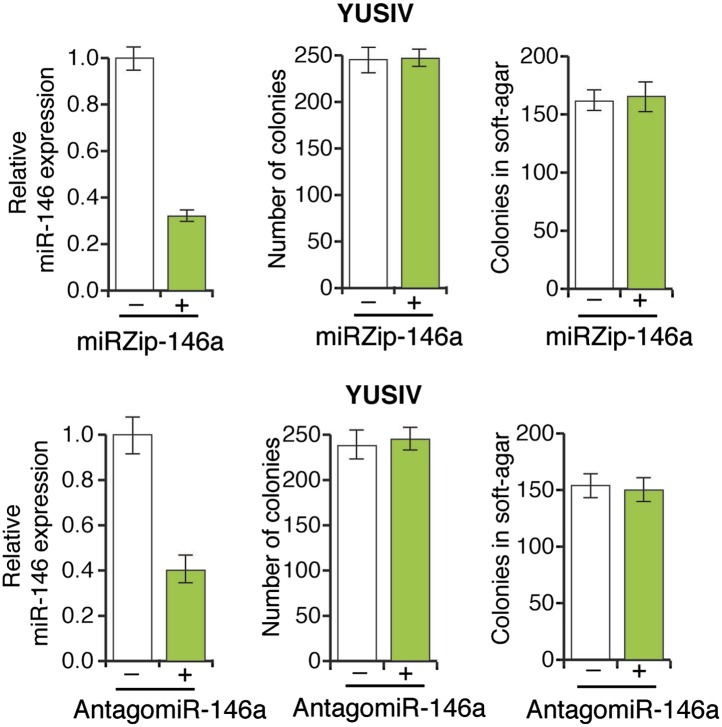
Inhibition of miR-146a expression does not block the proliferation and anchorage-independent growth of YUSIV cells. (Top panel) YUSIV cells either expressing a control miR-ZIP vector or miR-Zip-miR146a were analyzed for miR-146a expression (left), colony formation (middle) or growth in soft-agar (right). (Bottom Panel) YUSIV cells either expressing a control LNA-based control antagomiR or LNA-based miR-146a antagomiR were analyzed for miR-146a expression (left), colony formation (middle) or growth in soft-agar (right). **DOI:** http://dx.doi.org/10.7554/eLife.01460.020

**Figure 3—figure supplement 7.**
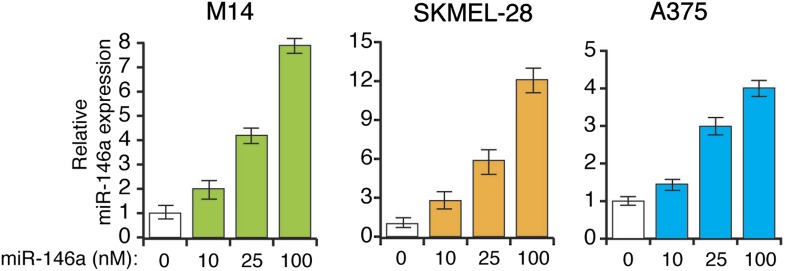
Analysis of miR-146a expression in indicated melanoma cell lines transfected with increasing concentration of synthetic miR-146a. qRT-PCR analysis of miR-146a expression in indicated melanoma cell lines. **DOI:** http://dx.doi.org/10.7554/eLife.01460.021

**Figure 3—figure supplement 8.**
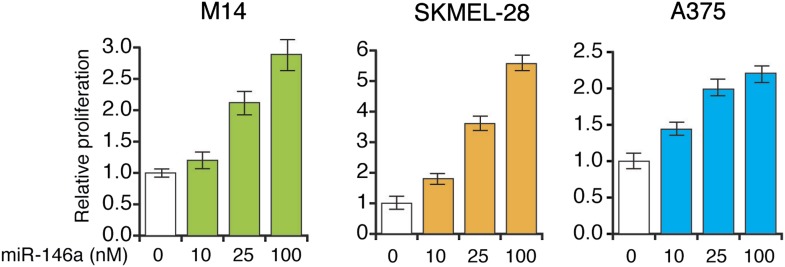
miR-146a enhances the growth of melanoma cell lines in a concentration dependent manner. Relative proliferation of indicated melanoma cell lines 5 days after the transfection of synthetic mature miR-146a at indicated concentrations. **DOI:** http://dx.doi.org/10.7554/eLife.01460.022

**Figure 3—figure supplement 9.**
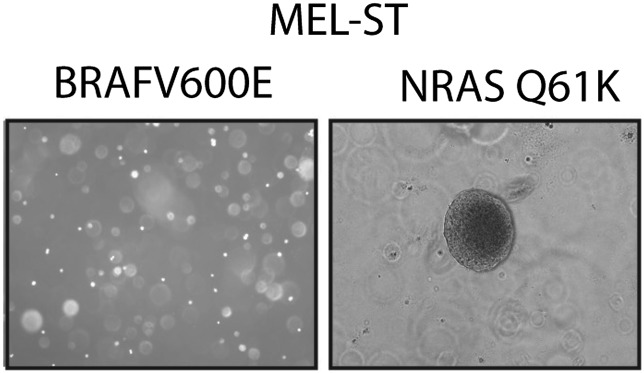
NRASQ61K is sufficient to transform MEL-ST cells. Representative images of soft-agar colonies formed by MEL-ST cells expressing BRAFV600E. NRASQ61K was used as a positive control. **DOI:** http://dx.doi.org/10.7554/eLife.01460.023

Finally, we subcutaneously injected SKMEL-28 cells stably expressing pre-miR-146a/C or pre-miR-146a/G, or an empty vector into immunocompromised mice. Although both pre-miR-146a/C and pre-miR146a/G enhanced tumor growth, the effect was much larger with pre-miR-146a/G (Figure 3H). To confirm that the stronger oncogenic effect of pre-miR-146a/G is due to increased abundance of mature miR-146a, we transfected SKMEL-28 cells with increasing amounts of synthetic miR-146a and monitored cell proliferation. Notably, increased levels of miR-146a enhanced proliferation in a dose-dependent manner (Figure 3—figure supplements 7 and 8). Collectively, our results show that the oncogenic activity of pre-miR-146a/G is greater than that of pre-miR-146a/C both in vitro and in vivo due to increased abundance of mature miR-146a.

Next, we asked whether miR-146a plays a role in BRAFV600E-mediated cellular transformation. For these experiments, we used immortalized but not transformed MEL-ST cells that can be transformed by a single oncogene. In agreement with a previous report (Chudnovsky et al., 2005), we found that BRAFV600E was not sufficient to transform the immortalized melanocytes (Figure 3—figure supplement 9). Activated alleles of the NRAS gene are the second most common oncogenic mutations in melanoma (Tsao et al., 2012). Therefore, we chose to analyze the role of miR-146a in the context of NRASQ61K-induced melanomagenesis. Similar to BRAFV600E, NRASQ61K transcriptionally activates miR-146a expression (Figure 1E). Notably, inhibition of miR-146a expression substantially reduced the ability of NRASQ61K-expressing cells to form colonies in soft-agar (Figure 3I) and tumors in mice (Figure 3J). Collectively, our results show that miR-146a promotes the initiation and progression of melanoma.

### miR-146a activates Notch signaling by downregulating NUMB

**Figure 4.**
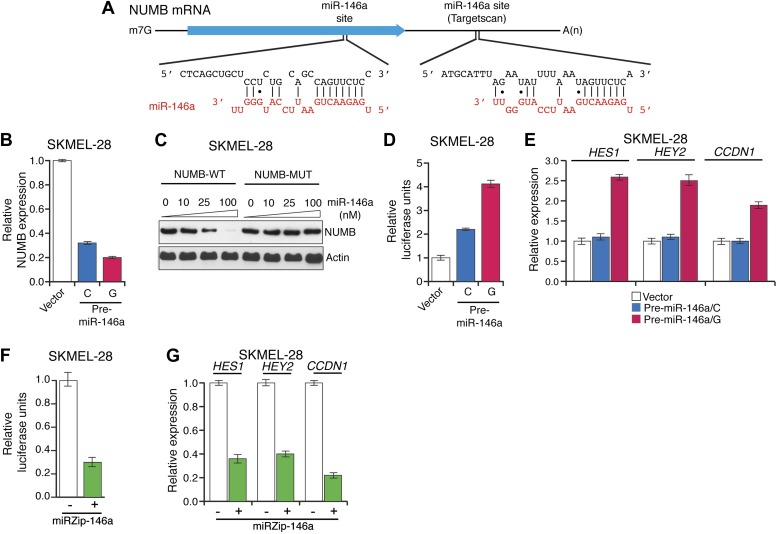
Downregulation of NUMB and activation of NOTCH signaling by pre-miR-146a/C and pre-miR-146a/G. (**A**) Schematic representation of the *NUMB* mRNA and potential miR-146a target sites in the coding region (blue arrow) and 3′-UTR. (**B**) qRT-PCR analysis of *NUMB* mRNA levels in SKMEL-28 cells expressing the indicated pre-miR-146a allele relative to the Vector control. (**C**) Western blot of NUMB expression in SKMEL-28 cells transfected with either wild-type *NUMB (NUMB-WT)* or miR-146a-resistant *NUMB (NUMB-MUT)* and increasing amount of synthetic miR-146a. (**D**) Dual luciferase assay using a CSL-Luciferase reporter to measure NOTCH activity in SKMEL-28 cells expressing the indicated pre-miR-146a alleles relative to the Vector control. (**E**) qRT-PCR analysis of the NOTCH targets *HES1, HEY2* and *CCDN1* mRNA in indicated samples. Actin mRNA was used as an internal control. (**F**) Dual luciferase assay using a CSL-Luciferase reporter to measure NOTCH activity in SKMEL-28 cells expressing miRZip-146a relative to the Vector control. (**G**) qRT-PCR analysis of *HES1, HEY2* and *CCDN1* mRNA in indicated samples. Actin mRNA was used as a loading control. **DOI:** http://dx.doi.org/10.7554/eLife.01460.024

To gain insight into the mechanism of miR-146a-mediated melanomagenesis and melanoma growth, we ectopically expressed pre-miR-146/C or pre-miR-146a/G in SKMEL-28 cells and performed microarray analysis. Of the genes downregulated by both pre-miR-146a/C and pre-miR-146a/G, TargetScan analysis identified 20 mRNAs with potential miR-146a binding sites in the 3′-UTR (Supplementary file 1A). We elected to focus on the *NUMB* mRNA because it encodes a repressor of NOTCH and several previous studies have shown that NOTCH functions as an oncogene in melanoma (Liu et al., 2006; Pinnix et al., 2009). We identified candidate miR-146a binding sites in both the 3′ UTR and coding region of *NUMB* mRNA (Figure 4A). Consistent with its increased oncogenic activity, pre-miR-146a/G decreased *NUMB* mRNA and protein levels more efficiently than pre-miR-146a/C (Figure 4B, Figure 4—figure supplement 1). Using a luciferase reporter assay, we found that miR-146a does not target the site within the NUMB 3′-UTR (Figure 4—figure supplement 2). By contrast, ectopic expression of miR-146a downregulated the expression of wild-type NUMB (NUMB-WT) open reading frame (ORF), whereas expression of a miR-146a-resistant NUMB ORF (NUMB-MUT) lacking the binding site in the coding region was unaffected (Figure 4C).

**Figure 4—figure supplement 1.**
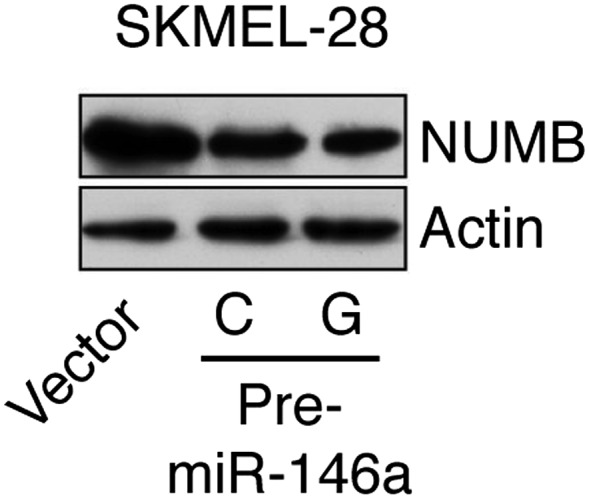
Ectopic expression of miR-146a downregulates NUMB protein expression. Immunoblot analysis of NUMB protein in SKMEL-28 cells stably transduced with empty vector, pre-miR-146a/C or pre-miR-146a/G. **DOI:** http://dx.doi.org/10.7554/eLife.01460.025

**Figure 4—figure supplement 2.**
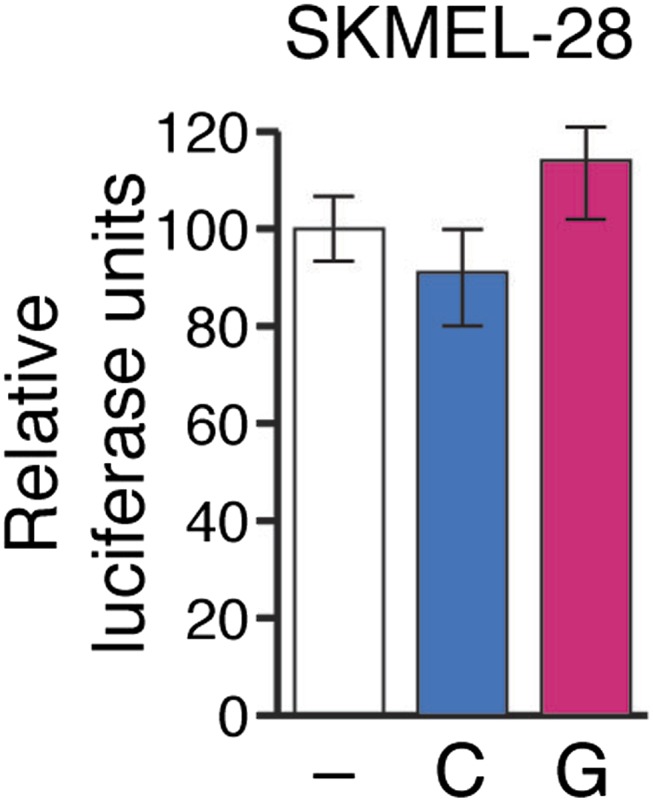
Relative luciferase activity of NUMB 3′UTR luciferase construct in SKMEL-28 cells expressing an empty vector (−), pre-miR-146a/C (C) or pre-miR-146a/G (G). **DOI:** http://dx.doi.org/10.7554/eLife.01460.026

To gain additional support for the idea that pre-miR-146a/G can activate Notch signaling more effectively than pre-miR-146a/C, we monitored NOTCH activity using a NOTCH responsive reporter plasmid CSL-pGL3-Luciferase (CSL-Luc) in SKMEL-28 cells expressing either pre-miR-146a/C or pre-miR-146a/G. Notably, both pre-miR-146a/C- and pre-miR-146a/G-stimulated CSL-Luc activity but again the effect was greater with pre-miR-146a/G (Figure 4D). Additionally, we analyzed expression of transcriptional target genes of Notch signaling. In agreement with the reporter assays, we found that Notch signaling targets were upregulated to a greater extent by pre-miR-146a/G compared to pre-miR-146a/C (Figure 4E). Conversely, stable knockdown of miR-146a decreased CSL-Luc activity and reduced Notch target gene expression (Figure 4F,G). Collectively, these results confirm that NUMB is a direct target of miR-146a and that miR-146a downregulates *NUMB* to activate Notch signaling.

### miR-146a-induced Notch signaling is necessary for the initiation and progression of melanoma

**Figure 5.**
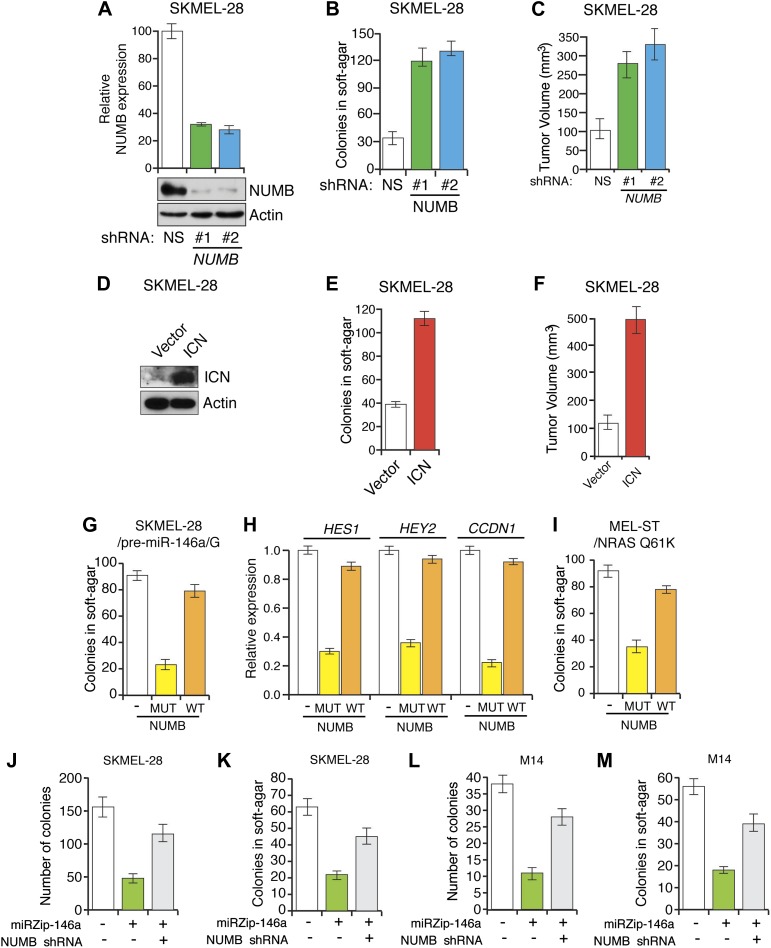
miR-146 oncogenic activity depends on the activation of the NOTCH signaling through downregulation of the tumor suppressor NUMB. (**A**) qRT-PCR analysis (top) and immunoblot (bottom) of *NUMB* expression levels in SKMEL-28 cells infected with two different shRNAs against NUMB relative to the control non-silencing shRNA (NS). (**B**) Number of colonies in soft-agar of SKMEL-28 cells expressing *NUMB* shRNAs relative to the control non-silencing shRNA (NS). (**C**) Average volume of tumors formed by SKMEL-28 cells (2.5 × 10^6^) expressing *NUMB* shRNAs, relative to the non-silencing shRNA (NS shRNA) control, injected subcutaneously into the flanks of nude mice (n = 5). (**D** and **E**) Immunoblot of NOTCH (**D**) and colony formation assay (**E**) of SKMEL-28 cells stably transduced with the activated intracellular NOTCH domain (ICN) or empty vector. (**F**) Average volume of tumors formed by SKMEL-28 cells expressing the activated intracellular NOTCH domain (ICN) relative to vector control. 2.5 × 10^6^ cells were injected subcutaneously into the flank of nude mice (n = 5). (**G**) Colony formation in soft-agar of SKMEL-28 cells expressing pre-miR-146a/G and transfected with either an empty vector, NUMB wild-type (WT) or an miR-146a-resistant NUMB (MUT). (**H**) qRT-PCR analysis of *HES1, HEY2* and *CCDN1* mRNA in indicated samples. Actin was used as an internal control. (**I**) Colony formation in soft-agar of MEL-ST/NRASQ61K transfected with either an empty vector, NUMB wild-type (WT) or miR-146a-resistant NUMB (MUT). (**J** and **K**) Number of colonies formed in liquid (**J**) or soft-agar (**K**) by SKMEL-28 expressing miRZip-146a (+) or the control miRZip (−) that either express a non-silencing shRNA or an shRNA against *NUMB*. Colonies were counted after 2 weeks (**J**) or 4 weeks (**K**) of growth. (**L** and **M**) Number of colonies formed in liquid (**L**) or soft-agar (**M**) by M14 expressing miRZip-146a (+) or the control miRZip (−) that either express a non-silencing shRNA or an shRNA against *NUMB*. Colonies were counted after 2 weeks (**L**) or 4 weeks (**M**) of growth. **DOI:** http://dx.doi.org/10.7554/eLife.01460.027

To investigate the role of NUMB downregulation and activated Notch signaling in melanoma cell growth, we knocked down *NUMB* in SKMEL-28 cells (Figure 5A) and monitored cellular proliferation in liquid culture, anchorage-independent colony formation in soft-agar and tumor formation in mice. Similar to ectopic expression of miR-146a, depletion of *NUMB* increased cellular proliferation (Figure 5—figure supplements 1 and 2), soft-agar colony formation (Figure 5B) and tumor formation in mice (Figure 5C). Finally, to test if increased NOTCH1 expression recapitulates NUMB loss, we ectopically expressed activated intracellular NOTCH1 (ICN) in SKMEL-28 cells (Figure 5D). As predicted, ectopic expression of ICN-enhanced cellular proliferation in liquid culture (Figure 5—figure supplements 3 and 4), colony formation in soft-agar (Figure 5E) and tumor formation in mice (Figure 5F). To confirm that NUMB downregulation is necessary for the ability of miR-146a to promote melanoma growth, we expressed either the miR-146a-resistant NUMB-MUT or NUMB-WT in SKMEL-28 cells expressing pre-miR-146a/G. We found that the expression of NUMB-MUT, but not NUMB-WT, prevented pre-miR-146a/G from promoting colony formation in soft-agar (Figure 5G) and activating NOTCH signaling (Figure 5H). Moreover, NUMB-MUT, but not NUMB-WT blocked the ability of NRASQ61K transformed MEL-ST cells to form colonies in soft-agar (Figure 5I). Notably, simultaneous antagonism of miR-146a and knockdown of NUMB restored proliferation of SKMEL-28 and M14 cells in both liquid culture and soft-agar (Figure 5J–M).

**Figure 5—figure supplement 1.**
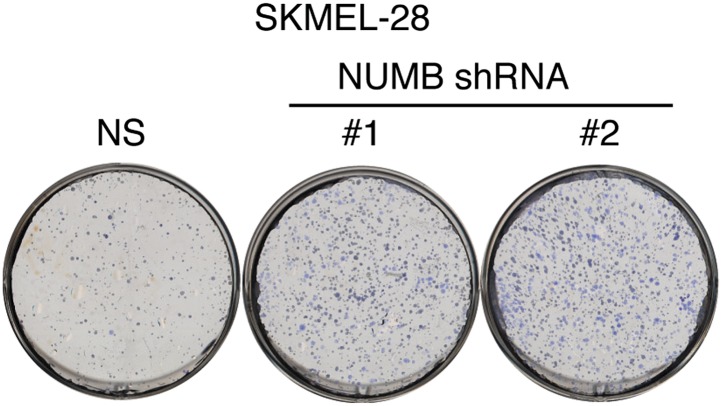
Inhibition of NUMB expression promotes growth of SKMEL-28 cells. Colony formation assay of SKMEL-28 cells expressing two different NUMB shRNAs relative to a non-silencing (NS) shRNA. **DOI:** http://dx.doi.org/10.7554/eLife.01460.028

**Figure 5—figure supplement 2.**
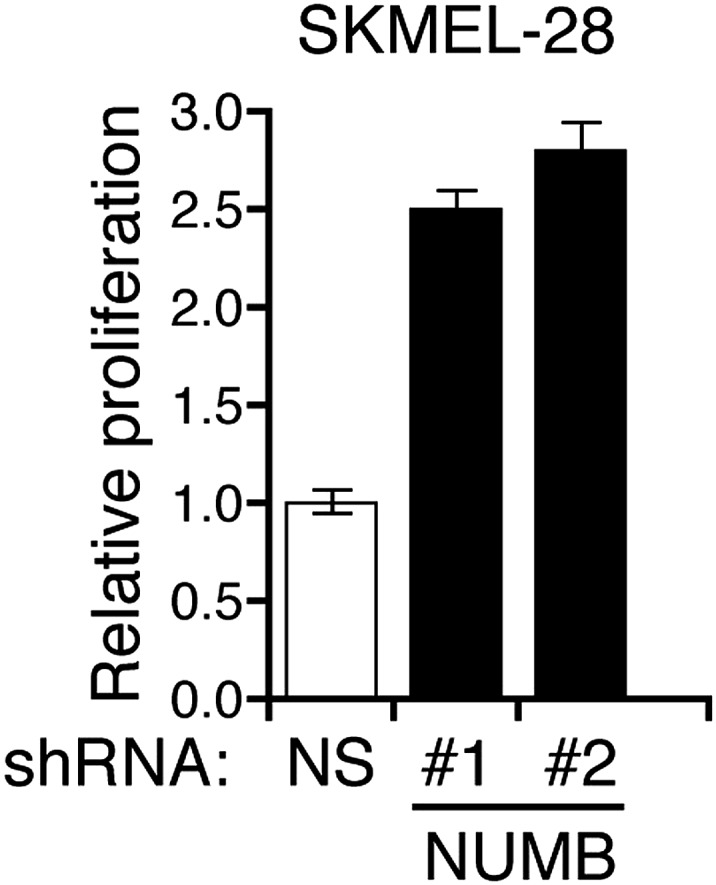
Inhibition of NUMB promotes proliferation of melanoma cells. Proliferation assay of SKMEL-28 cells expressing two different NUMB shRNAs relative to a non-silencing (NS) shRNA. **DOI:** http://dx.doi.org/10.7554/eLife.01460.029

**Figure 5—figure supplement 3.**
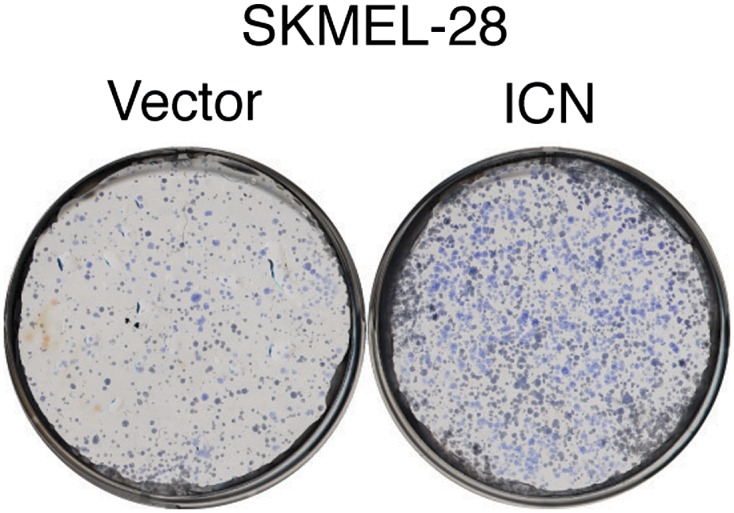
Ectopic expression of activated Notch promotes the growth of melanoma cells. Colony formation assay of SKMEL-28 cells stably transduced with the activated intracellular NOTCH domain (ICN) or empty vector. **DOI:** http://dx.doi.org/10.7554/eLife.01460.030

**Figure 5—figure supplement 4.**
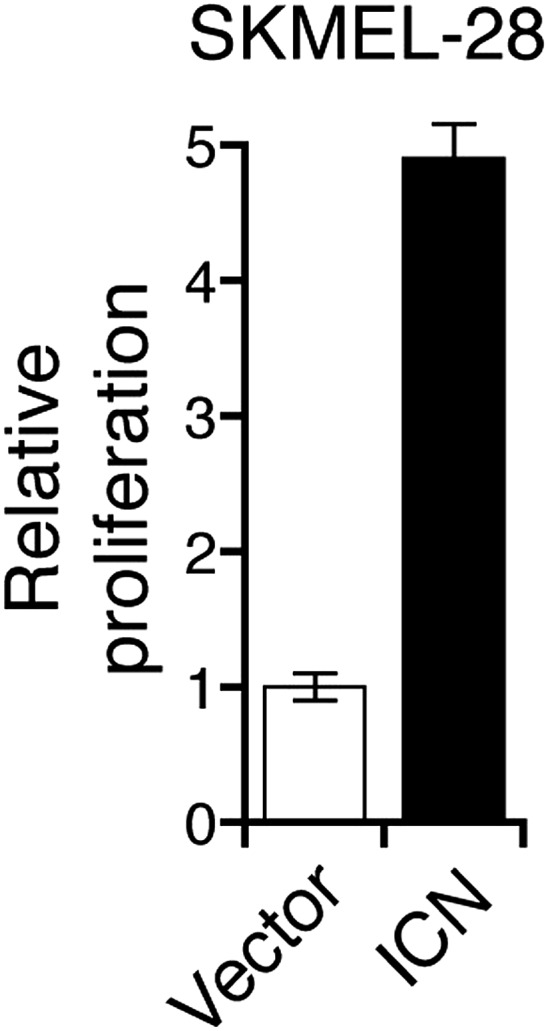
Ectopic expression of intracellular Notch promotes proliferation of melanoma cells. Proliferation rate of SKMEL-28 cells stably expressing Intracellular NOTCH (ICN) relative to cells with empty vector. Proliferation rate was measured after 72 hr of growth using a colorimetric MTT assay. **DOI:** http://dx.doi.org/10.7554/eLife.01460.031

**Figure 6.**
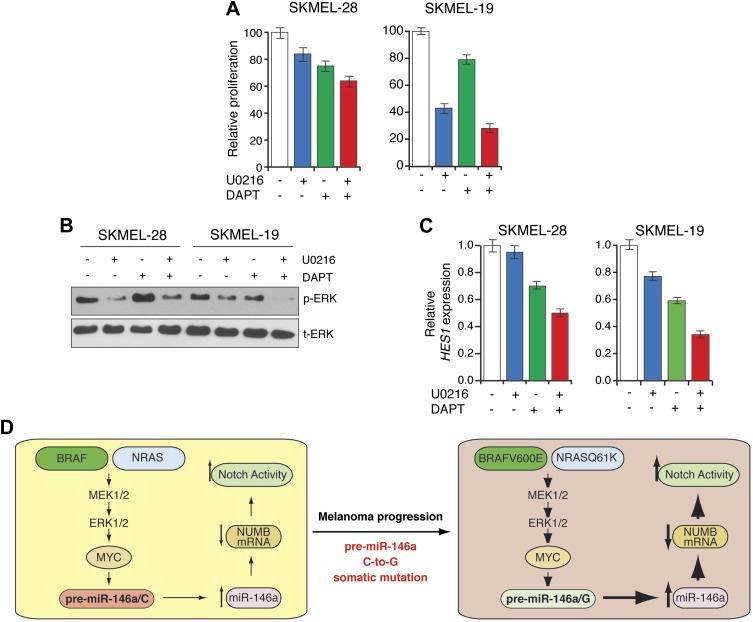
Synergistic melanoma growth inhibition by simultaneous blockage of Notch and BRAF signaling. (**A**) MTT proliferation assay. (**B**) Immunoblot analysis of phosphorylated (p-) ERK and total (t-) ERK and (**C**) qRT-PCR analysis of NOTCH target *HES1* in SKMEL-28 and SKMEL-19 cells left untreated or treated with U0216, DAPT or combination of both drugs. (**D**) Model. **DOI:** http://dx.doi.org/10.7554/eLife.01460.032

The results described above suggested that melanomas with elevated miR-146a expression might be sensitive to combined inhibition of BRAF-MEK-ERK and Notch signaling. To test this idea we treated BRAF mutant SKMEL-28 and SKMEL-19 cells, with the MEK inhibitor U0216, the gamma secretase inhibitor DAPT, or both drugs. Notably, combined U0216 and DAPT treatment inhibited proliferation of SKMEL-28 and SKMEL-19 cells much strongly than either of the drugs alone (Figure 6A). As expected, U0216 efficiently blocked MAPK signaling (Figure 6B) and DAPT inhibited the transcriptional targets of Notch signaling (Figure 6C). These results further establish the role of miR-146a in activating Notch signaling. Collectively, these results indicate that the ability of miR-146a to block NUMB expression and activate Notch signaling is necessary for miR-146a to promote melanoma initiation and progression.

### A somatic oncogenic mutation in pre-miR-146a becomes enriched during melanoma progression

To investigate the clinical relevance of our findings, we cloned and sequenced pre-miR-146a from the genomic DNA of three independent melanocyte cultures and 18 established melanoma cell lines or short-term melanoma cultures. We found that two out of three melanocytes were homozygous for pre-miR-146a/C and one was a pre-miR-146a/C:pre-miR-146a/G heterozygote. By contrast, all melanoma cell lines were either pre-miR-146a/C:pre-miR-146a/G heterozygotes or pre-miR-146a/G homozygotes (Table 1).

**Table 1.** Sequence at position 40 of *pre-miR-146a* in primary human melanocytes, human melanoma cell lines and clinical samples **DOI:** http://dx.doi.org/10.7554/eLife.01460.033

Human melanoma cell lines	
Cell Line	
Melanocytes-1	CC
Melanocytes-2	CC
Yale SPORE melanocytes	C***G***
WM3918	C***G***
YUHEF	C***G***
YUVON	C***G***
YUSIV	***GG***
YUROB	***GG***
YUROL	C***G***
MeWo	***GG***
UCC257	C***G***
SKMEL28	C***G***
A375	***GG***
SKMEL5	***GG***
M14	C***G***
SKMEL19	***GG***
YULAC	***GG***
YUGEN8	***GG***
YUSAC2	***GG***
YURIF	C***G***
SKMEL103	C***G***

Matched clinical melanoma samples (Nevus/Primary)		
Sample	Nevus (Type)*	Primary Melanoma
1	C***G*** (IM)	C***G***
2	CC (C)	C***G***
3	C***G*** (C)	***GG***
4	C***G*** (C)	***GG***
5	C***G*** (LJ)	C***G***
6	C***G*** (IM)	C***G***
7	C***G*** (IM)	C***G***
8	C***G*** (LJ)	C***G***
9	C**G (**IM**)**	C**G**
10	CC (C)	C**G**

Matched Clinical Melanoma Samples (Primary/Metastatic)		
Sample	Primary Melanoma	Metastatic Melanoma
1	C***G***	***GG***
2	C***G***	C***G***
3	C***G***	***GG***
4	C***G***	C***G***
5	CC	***GG***
6	C***G***	***GG***
***7***	*C****G***	***GG***
8	CC	CC
9	C**G**	C**G**
10	C**G**	C**G**
11	CG	C**G**
12	CG	**GG**
13	CG	**GG**
14	CG	**GG**

* IM, Intradermal melanocytic nevus. C, Compound nevus. LJ, Lentigenous juctional nevus. Samples highlighted in gray indicate C-to-G mutation during melanoma progression.

Next, we sequenced 48 melanoma samples consisting of 10 matched pairs of melanocytic nevi and primary melanomas, as well as 15 matched pairs of primary and metastatic melanomas (Table 1; Supplementary file 1B,C). As shown in Table 1, in 4 of 10 cases the nevus contains a pre-miR-146a/C allele that is a pre-miR-146a/G allele in the matched primary melanoma. Remarkably, in 8 of 15 cases analyzed, a pre-miR-146a/C allele in the primary melanoma is a pre-miR-146a/G allele in the matched metastases. In one case, both pre-miR-146a/C alleles in the primary melanoma are pre-miR-146a/G alleles in the matched metastasis. Finally, we also analyzed a publically available melanoma SNP datasets (Gast et al., 2010) and also observed an enrichment for pre-miR-146a/G during melanoma progression (Supplementary file 1D). Collectively, these results reveal a selection for pre-miR-146a/G during melanoma progression. Moreover, the results with matched patient samples indicate that enrichment of pre-miR-146a/G results from a C-to-G mutation in pre-miR-146a.

## Discussion

In this report, we demonstrate a critical role for miR-146a in the initiation and progression of BRAF/NRAS-positive melanomas, which is summarized in Figure 6D and discussed below. In addition, our results reveal a pharmacologically tractable pathway for the treatment of melanoma.

We identified miR-146a as the microRNA whose expression was most upregulated by activated BRAF. Upregulation of miR-146a by activated BRAF, as well as activated NRAS, occurs through the MAPK signaling pathway. Accordingly, we find that BRAF and NRAS mutant melanoma cell lines and short-term melanoma cultures show higher levels of miR-146a compared to those that are wild type for these genes.

A major function of the MAPK pathway is to activate transcription by regulating the stability and expression of multiple transcription factors primarily through direct phosphorylation (Qi and Elion, 2005). We show that the MAPK pathway regulates the phosphorylation of the transcription factor MYC, which in turn binds to the promoter of miR-146a and stimulates its transcription. Notably, MYC has been found to stimulate transcription of several other miRNAs (Chang et al., 2008). For example, MYC has been shown to directly activate transcription of the oncogenic miR-17-92 cluster and thereby promote cell proliferation, survival, angiogenesis, and metabolic reprogramming in a number of tumor cell lines (Mu et al., 2009; Dews et al., 2010).

miRNAs and components of miRNA biogenesis pathways such as Dicer have been implicated in several aspects of melanocyte biology as well as in melanoma initiation and progression (Levy et al., 2010; Bonazzi et al., 2012). Previous studies have shown that miR-146a can function either as an oncogene or as a tumor suppressor depending upon the cell type (Boldin et al., 2011; Chen et al., 2013). For example, miR-146a has been shown to function as an oncogene in a variety of human cancers including papillary thyroid carcinoma (PTC), triple negative sporadic breast cancers and anaplastic thyroid carcinoma (Jazdzewski et al., 2008; Pacifico et al., 2010; Garcia et al., 2011).

miRNAs function primarily by targeting mRNAs and either promoting their degradation or blocking their translation (He and Hannon, 2004). Our analysis identified 20 potential targets of miR-146a, including NUMB, which is a well-characterized Notch signaling inhibitor. It is thought that NUMB negatively regulates NOTCH, potentially through a direct protein–protein interaction that requires the phosphotyrosine-binding (PTB) domain of NUMB and either the RAM23 region or the very C-terminal end of NOTCH (Guo et al., 1996). Consistent, with our results, miR-146a was previously reported to target NUMB mRNA in mouse myocytes C2C12 cells (Kuang et al., 2009). We confirmed NUMB as a direct target of miR-146a and showed that NUMB targeting and activation of Notch signaling was necessary for the ability of miR-146a to promote the initiation and progression of melanoma.

Many cancers, including melanoma, have been found to have increased Notch signaling, which results from two distinct mechanisms (Ranganathan et al., 2011). First, gain-of-function mutations in NOTCH1 can increase Notch signaling (Ranganathan et al., 2011). Second, decreased expression of NUMB can result in increased Notch signaling (Guo et al., 1996). For example, NUMB expression is decreased in breast and lung cancer and NUMB is considered to be a tumor suppressor in these malignancies (Lindsay et al., 2008). In this study, we have shown that Notch signaling can also be increased by upregulation of a miRNAs that targets NUMB.

Several previous studies have shown that pre-miR-146a contains a single nucleotide polymorphism (SNP) (C>G rs2910164), which has been associated with various aspects of tumor biology (Jazdzewski et al., 2008; Hezova et al., 2012; Hung et al., 2012; Lung et al., 2012; Wang et al., 2012; Yamashita et al., 2013). We found that the oncogenic activity of pre-miR-146a/G is substantially greater than that of pre-miR-146a/C, which results, at least in part, from more efficient processing of pre-miR-146a/G compared to pre-miR-146a/C. Our results are consistent with a previous report showing that pre-miR-146a/G undergoes a more efficient nuclear processing compared to pre-miR-146a/C and thus give rise to a higher amount of mature miR-146a (Jazdzewski et al., 2008).

Most importantly, our analysis of melanoma cell lines and matched patient samples of nevi, primary, and metastatic melanoma reveal enrichment of the more oncogenic variant, pre-miR-146a/G, during melanoma progression. Our results are consistent with previous studies that observed enrichment for pre-miR-146a/G (Gast et al., 2010; Supplementary file 1D) and increased miR-146a expression (Philippidou et al., 2010) during melanoma progression.

Finally, our findings have important therapeutic implications. Both the Notch and BRAF-MEK-ERK signaling pathways are amenable to pharmacological inhibition (Rizzo et al., 2008; Bollag et al., 2010; Flaherty et al., 2010). Our results suggest that combining clinically approved γ–secretase inhibitors, which block Notch activation, with BRAF inhibitors, which block BRAF-MEK-ERK signaling, might be a more effective treatment of BRAF/NRAS mutant melanomas.

## Materials and methods

### Preparation of small RNA library and deep sequencing

Small RNAs isolated from Vector-infected, BRAFV600E-infected and quiescent cells were cloned essentially as described (Ambros et al., 2003). The Firecrest, Bustard and Gerald analysis module (Illumina) were used for image analysis, base calling and filtering the raw data (36 bp reads) from each run to generate the sequence reads. Sequences were further filtered to those containing 18–30 bp followed by the first 6 bp of the 3′ linker (CTGTAG). Sequences that passed filters were clustered and mapped to known human microRNAs using BLAST. The Fisher exact test was used to identify differentially-expressed miRNAs. The odds ratio and 95% confidence interval were computed for each treatment using the ‘fisher test’ function in R 2.7.0 based on conditional maximum likelihood estimation. Adjusted p values were obtained using the Benjamini–Hochberg method to correct for multiple comparisons, and miRNAs with adjusted p value<0.05 were considered significant. Deep sequencing results were submitted to Gene Expression Omnibus (Accession No. GSE39983).

### Cell culture, plasmids, shRNAs, lentivirus preparation and luciferase assay

WI-38 and IMR-90 cells were purchased from ATCC and grown as recommended. Different primary human melanocyte cultures were purchased from Lonza, Invitrogen, and Yale SPORE in Skin Cancer. Melanoma cell lines were purchased from ATCC and grown as recommended. All short-term melanoma cultures were purchased from Yale Skin SPORE (Yale University) and grown as recommended. The plasmids pBabe puro-BRAFV600E (15269; Addgene, Cambridge, MA), pBabe puro-HRAS V12 (15269; Addgene), pBabe puro-HRAS V12 S35 (12274; Addgene), pBabe puro-MEK-DD (15268; Addgene), and EF.hICN1.Ubc.GFP (17626; Addgene) were purchased from Addgene. FG12-NRAS 61K was a kind gift of Maria Soengas and Mikhail A Nikiforov. pre-miR146a/C, pre-miR146a/G were cloned into the lentiviral expression vector FG12-CMV. A 1081 bp fragment of the NUMB 3′UTR, including miR146a seed sequence, was PCR amplified by using XhoI-NotI primers (Supplementary file 1E) and cloned into psiCHECK-2 luciferase vector. NUMB coding sequence (CDS) was PCR amplified by using HindIII-NotI primers (Supplementary file 1E) and cloned into pcDNA3.1-Hygro plasmid. The miR146a seed sequence was mutated by using specific primers listed in Supplementary file 1E 5 and the QuikChange XL Site-Directed Mutagenesis Kit (Agilent Technologies, Santa Clara, CA) by following manufacturer’s instruction. *MYC*, *NUMB, ETS1, ELK1, c/EBPβ and RelA (p65 subunit of NF-κB)* shRNAs in the pLKO.1 lentiviral expression vector were obtained from Open Biosystems. The anti-miR-146a vector miRZip-146a was obtained from System Biosciences. For viral particles production viral expression vectors and viral packaging plasmids were co-transfected into 293T cells using Effectene (Qiagen, Valencia, CA) as per manufacturer’s recommendations. Purified virus particles were infected into primary or melanoma cell lines, and cell lines stably transduced with viral DNA were selected by growth on puromycin or by sorting GFP-positive cells using a flow cytometer. To monitor NOTCH activity SKMEL28 cells stably expressing pre-miR-146a/C, pre-miR-146a/G or empty vector were transfected with a NOTCH responsive reporter plasmid CSL-pGL3-Luciferase (CSL-pGL3-Luc). After 48 hr cells were lysed and luciferase activity was measured by using Dual-Luciferase reporter assay system (Promega, Madison, WI).

### RNA preparation, cDNA preparation, quantitative PCR analysis and ChIP assays

For mRNA expression analyses, total RNA was extracted with TRIzol (Invitrogen) and purified using RNAeasy mini columns (Qiagen), and cDNA was generated using M-MuLV first-strand cDNA synthesis kit (New England Biolabs) as per manufacturer’s instructions. For miR-146a expression analyses, total RNA was prepared using TRIzol (Life Technologies, Grand Island, NY), small RNAs were enriched using the miRVana kit (Ambion), and cDNA was prepared using the miScript Reverse Transcription Kit (Qiagen) as per manufacturer’s instructions. Quantitative RT-PCR was performed using Power SYBR-green kit (Applied Biosystems, Foster City, CA) for mRNA expression analysis or the miScript SYBR-green PCR assay kit (Qiagen), as per manufacturer’s instructions. GAPDH was used as an internal control. ChIP experiments were performed as described previously (Raha et al., 2005). MYC binding to the miR-146a promoter and a negative control region was determined using the primers listed in Supplementary file 1E.

### Antibodies and immunoblot analysis

Whole cell protein extracts were prepared using IP lysis buffer (Pierce) containing Protease Inhibitor Cocktail (Roche, Madison, WI) and Phosphatase Inhibitor Cocktail (Sigma–Aldrich, St. Louis, MO). Protein concentration was estimated using a Bradford Assay kit (Bio-Rad). Proteins separated on 10% or 12% polyacrylamide gels were transferred to PVDF membranes using a wet transfer apparatus from Biorad. Membranes were blocked with 5% skim milk and probed with primary antibodies followed by the appropriate secondary HRP-conjugated antibody (GE healthcare, UK). Blots were developed using the Supersignal Pico Reagent (Pierce, Rockland, IL). Antibody information is provided in Supplementary file 1E.

### Patient samples collection: matched samples of nevi, primary melanoma and metastatic melanoma

This study was approved by Boston University School of Medicine, Institutional Review Board (IRB docket #H-29789 and H-29979) and University of Massachusetts Medical School, Instiutional Review Board (IRB docket# H00001007). Archived tissue with a diagnosis of primary cutaneous malignant melanoma and nevus from the same patient (n = 10) and primary cutaneous malignant melanoma and metastases from the same patient (n = 15) were retrieved from the pathology files of the Skin Pathology Laboratory, Boston University School of Medicine, Boston, MA, USA and University of Massachusetts Medical School, Worcester Histopathologic sections of all cases were reviewed by two board-certified dermatopathologists (initial sign-out on all by a dermatopathologist; cases were then re-reviewed, and the diagnoses were confirmed by MM). All patient data were de-identified. The informed consent was not required because all the samples used in study were archival tissues.

### Colony formation assay, soft-agar assay and tumorigenesis assay

For soft-agar and colony formation assays, individual cell lines were seeded in triplicates at three different dilutions, ranging from 1 × 10^3^ to 1 × 10^4^ cells. For soft-agar assays, cells were seeded into a 0.35% soft-agar layer. Each experiment was repeated at least twice. Colonies were stained with 0.005% crystal-violet solution and counted after 4 weeks. Athymic nude (NCr nu/nu) mice (8 week old) were injected subcutaneously with SKMEL-28 or M14 cell lines expressing pre-miR146a/C or /G (2.5 × 10^6^ cells), miRZip-146a or transduced with the control construct. For melanomagenesis experiments, athymic nude (NCr nu/nu) mice (8 week old) were injected with 1.0 × 10^6^ NRASQ61K transformed MEL-ST cells expressing a control construct or miRZip-146a. Tumor volume was calculated using the formula: length × width^2^ × 0.5.

### Sequencing of pre-miR-146a SNP and genotyping of BRAF and NRAS genes

Genomic DNA was isolated either from the indicated cell lines or from formalin-fixed paraffin-embedded nevi, primary or metastatic melanoma patient samples. For melanoma cell lines, cell pellets were incubated at 50°C overnight in 100 mM NaCl, 10 mM Tris–HCl, pH8, 25 mM EDTA, 0.5 SDS and 0.1 mg/ml Proteinase K and the genomic DNA was extracted with phenol-chloroform. For paraffin-fixed tissues, tissue sections scraped from five 10-μm samples were incubated at 60°C overnight (in SSC buffer, 180 mM NaCl, 0.45% SDS, 2 mg/ml Proteinase K and 1 mM DTT) and the DNA was extracted with phenol–chloroform. A 227 bp fragment including pre-miR146a was amplified by PCR using the primers listed in Supplementary file 1E 5, cloned using pGEM-T kit (Promega), and plasmid DNA isolated from 24 bacterial colonies were sequenced. Similarly, fragments of BRAF and NRAS were amplified for genotyping using primers listed in Supplementary file 1E from genomic DNA isolated from patient-derived samples, cloned using pGEM-T kit (Promega), and plasmid DNA isolated from 24 bacterial colonies were sequenced using S6 primers. All the sequencing was performed using Sanger sequencing method that typically have the error rate that range from 0.001 to 1% (Hoff, 2009).

### Microarray experiments, data analysis and melanoma SNP data analyses

For microarray experiments, total RNA isolated from SKMEL-28 cells transduced by pre-miR-146a/C or /G or the Vector control was used to generate labeled antisense RNA using the Ambion MessageAmp Kit and hybridized to Illumina HumanHT-12 V4.0 expression beadchip using Illumina’s protocol. The microarray data were processed using GenomeStudio (Illumina), log2-transformed, and quantile-normalized using the ‘lumi’ package of Bioconductor. All samples passed quality-control (QC) assessment, which included checking various control plots as suggested by Illumina as well as other standard microarray-related analyses. Differential expression analyses were performed using the ‘limma’ package, and a moderated t-test with a Benjamini–Hochberg multiple testing correction procedure was used to determine statistical significance (adjusted p<0.05). Pathway analysis of differentially expressed genes for each comparison was performed using MetaCore (version 6.8 build 29806; GeneGo). Microarray data were submitted to Gene Expression Omnibus (Accession No. GSE39294). For analyzing the SNP dataset the CEL files were downloaded from gene expression omnibus and genotype callings by the CRLMM method on the data of GSE17534 and GSE7822 were done by the R program ‘crlmm’ in the BioConductor ‘oligo’ package (Scharpf et al., 2011). The cell line stages of GSE17534 dataset was extracted from Table 1 of the previously published study (Gast et al., 2010).

### Statistical analysis

All the experiments were done at three different times using independent sample preparation in triplicate. Mean values for individual experiments are expressed as mean ± SEM.

## Additional files

Supplementary file 1. (**A**) List of genes down-regulated by both pre-miR-146a/C and pre-miR-146a/G with miR-146a seed sequences (by TargetScan). (**B**) Genotyping of patient samples for BRAF and NRAS mutations. (**C**) Mutant BRAF, mutant NRAS and miR-146a C and G allele frequencies. (**D**) Melanoma SNP dataset analyses for pre-miR-146a/C and pre-miR-146a/G frequencies. (**E**) Primer sequences for qRT-PCR analysis; clone ID and catalog numbers for shRNAs (Open Biosystems); antibodies used; source and concentration of chemical inhibitors used. **DOI:** http://dx.doi.org/10.7554/eLife.01460.034

### Major datasets

The following datasets were generated:

ForloniM, DograS, DongY, ConteD, MahalingamM, GreenM, WajapeyeeN, 2013, Oncogenic BRAF activated miR-146a functions as an oncogene, ; http://www.ncbi.nlm.nih.gov/geo/query/acc.cgi?token=cvmrgsssjxmlhat&acc=GSE39294, Publicly available at GEO (http://www.ncbi.nlm.nih.gov/geo/).

ForloniM, DograS, DongY, ConteD, ZhuJ, MahalingamM, GreenM, WajapeyeeN, 2013, Oncogenic BRAF activated miR-146a functions as an oncogene [miRNA-seq], ; http://www.ncbi.nlm.nih.gov/geo/query/acc.cgi?token=glitmgwadhsxtep&acc=GSE39983, Publicly available at GEO (http://www.ncbi.nlm.nih.gov/geo/).
